# Proteins acting out of (dis)order

**DOI:** 10.7554/eLife.32762

**Published:** 2017-11-21

**Authors:** David Eliezer

**Affiliations:** Department of BiochemistryWeill Cornell Medical CollegeNew YorkUnited States

**Keywords:** allostery, frustration, disorder, None

## Abstract

A disordered region at the N-terminus of the glucocorticoid receptor can fine tune how cells respond to a hormone via an allosteric mechanism.

**Related research article** Li J, White JT, Saavedra H, Wrabl JO, Motlagh HN, Liu K, Sowers J, Schroer TA, Thompson EB, Hilser VJ. 2017. Genetically tunable frustration controls allostery in an intrinsically disordered transcription factor. *eLife* **6**:e30688. doi: 10.7554/eLife.30688

For a long time it was thought that proteins only worked after folding into specific three-dimensional shapes (Anfinsen, 1973). However, disordered proteins – flexible proteins that lack a well-defined shape or structure – are found in almost all organisms, and our understanding of how protein disorder is coupled to protein function continues to evolve.

Disordered proteins can bind to other well-folded proteins and fold around them, thus gaining structure while executing their functions. Alternatively, disordered proteins can attach to other proteins via short, typically linear motifs, influencing the target protein while remaining largely flexible and unstructured (Mittag et al., 2010; Babu et al., 2012; Van Roey et al., 2014). However, it was commonly assumed that disordered proteins were unable to exhibit allostery, or “action at a distance”. Now, in eLife, Vincent Hilser and colleagues at Johns Hopkins University and the University of Houston report that disordered proteins can show allostery, and that alterations in the disordered regions of a protein can tune this effect to regulate the protein’s function (Li et al., 2017).

Allostery involves a change made at one location in a protein (the so-called allosteric site) exerting a functional effect at a distant region of the same protein (the functional site). Intuitively, we like to think of allostery as being transmitted via a physical connection between the allosteric site and the functional site: that is, pulling or pushing at the initial site is ‘felt at the distant site. In order for such a signal to be transmitted, we also assume that the connection between the sites must be rigid (Figure 1A and B). If the connection is instead flexible and dynamic, as in a disordered protein, it would seem that perturbation at one site cannot be effectively communicated to a distant location. As is often the case, however, our intuition based on the macroscopic physical world can lead us astray when considering things on the molecular scale.

**Figure 1.**
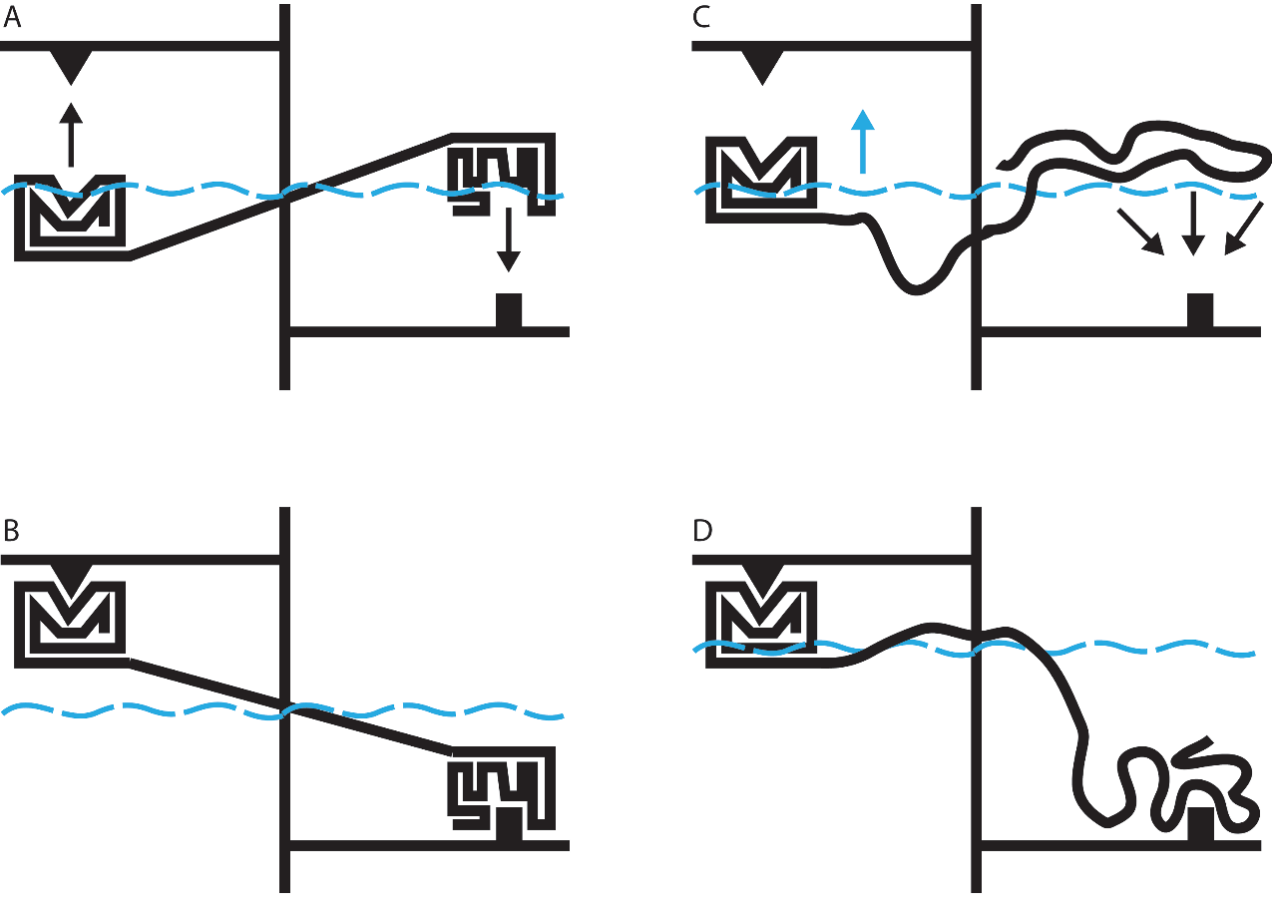
Allosteric coupling in well-structured and flexible proteins. (**A, B**) Cartoon illustrations of two rigidly linked well-structured domains in an assumed mechanical model. The allosteric site is on the right, above its ligand (black downward arrow), and the functional site is on the left, below its binding partner (black upward arrow). Binding at the allosteric site pivots the rigid linker around a fulcrum; this applies a force to the domain containing the functional site, which binds to its partner. (**C**) Cartoon illustration of allosteric coupling between a structured domain (left) and a disordered domain (right). The flexibility of the disordered region means that a force is not directly applied to the structured domain when the disordered region binds to its binding partner (black downward arrows). (**D**) Nevertheless, binding of the disordered region alters the energetics of the system in a way that makes binding at the functional site more favorable. In this illustrative case, we simulate these energetics by placing our mechanical system in a water bath (blue dashed line). By binding to its ligand (bottom right), the disordered domain raises the water level in the bath (blue upward arrow in C), indirectly causing the domain containing the functional site to float closer to its target. In real proteins, the energetic coupling is mediated by the rebalancing of the ensemble of different possible states that occurs when the allosteric site binds to its ligand (see Hilser and Thompson, 2007).

Hilser’s earlier work has shown that it may be difficult or impossible to understand the behavior of a protein in the context of a single direct link between sites of action and effect. Instead, it must be understood in terms of a constantly changing ensemble of different protein structures, in which changes in the stability of one site influence the entire ensemble in a way that can alter the likelihood of changes at a distant site (Hilser et al., 1998). The beauty of this theory is that it applies equally to well-structured proteins and to disordered proteins. Indeed, over 10 years ago, Hilser used this approach to predict that disordered protein regions could exert allosteric effects on other protein domains (Figure 1C and D; Hilser and Thompson, 2007).

Since this original prediction, a handful of high profile reports have documented allostery in disordered proteins, including in the oncoprotein E1A (Ferreon et al., 2013), and in a bacterial antitoxin (Garcia-Pino et al., 2010). Now, Hilser and colleagues, including Jing Li as first author, have focused on a protein known as the glucocorticoid receptor, a critical transcription factor that controls how cells respond to steroid hormones. Under different conditions, cells will produce versions of this receptor that differ only in the length of a disordered region at one end of the protein. Li et al. show that these different forms of the glucocorticoid receptor also alter the strength of the hormonal response to differing extents. This is possible because the disordered region enhances the hormonal response by enhancing DNA binding at a site located in an entirely different part of the protein. Paradoxically, the same disordered region also inhibits the hormonal response by directly regulating another domain, the F-domain. Depending on the specific form of the N-terminal disordered protein region, the sum of these two opposing effects is different, allowing the cell to fine tune its response to the presence of the hormone.

These findings imply that disordered proteins may have certain advantages for allosteric regulation compared with well-structured proteins. Producing different forms of well-structured proteins is challenging, because splicing new protein segments into an existing structure, or excising segments out of one, may be difficult to do while maintaining the protein’s architecture. In contrast, disordered proteins are essentially unconstrained by structure. As such, they can easily tolerate the insertion or removal of segments to generate distinct forms (Romero et al., 2006). Li et al. show that cells producing distinct forms of a specific disordered protein region, featuring different allosteric properties, exhibit different hormonal responses. More broadly, their work implies that altering a disordered region of a protein is a particularly flexible approach by which cells and organisms can fine tune allosteric regulation of critical biological processes. It can therefore be expected that this mechanism will prove to be widespread and important throughout biology.
